# Dental caries experience and determinants in young adults of the Northern State Medical University, Arkhangelsk, North-West Russia: a cross-sectional study

**DOI:** 10.1186/s12903-017-0426-x

**Published:** 2017-11-28

**Authors:** Sergei N. Drachev, Tormod Brenn, Tordis A. Trovik

**Affiliations:** 1 Department of Community Medicine, Faculty of Health Sciences, UiT The Arctic University of Norway, N-9037 Tromsø, Norway; 2 International School of Public Health, Northern State Medical University, Troickij av. 51, NSMU, ISPHA, office 1252, 163061 Arkhangelsk, Russia

**Keywords:** DMFT, Young adults, Medical and dental students, North-West Russia

## Abstract

**Background:**

Little information exists about the experience of and risk factors for dental caries in young adults in Russia. We investigated dental caries experience and determinants in medical and dental students in North-West Russia.

**Methods:**

This cross-sectional study included 442 medical and 309 dental undergraduate students of Russian nationality aged 18–25 years from the Northern State Medical University, Arkhangelsk, Russia. Information on socio-demographic factors and oral health behaviour (regularity of dental visits, frequency of tooth-brushing, using toothpaste with fluoride, and skipping tooth-brushing) was obtained from a structured, self-administered questionnaire. Dental caries experience was based on the decayed (D) missing (M) filled (F) teeth (T) index and the Significant Caries (SiC) index, which were assessed through dental examination. Students with a DMFT index ≥9 were placed in the SiC group. Negative binomial hurdle and multivariable binary logistic regressions were used for statistical analyses.

**Results:**

The prevalence of dental caries (DMFT >0) was 96.0%, overall mean DMFT index was 7.58 (DT: 0.61, MT: 0.12, and FT: 6.84), and the corresponding SiC index was 12.50. Age 21–25 years (incidence rate ratio [IRR] = 1.09, 95% confidence interval [CI]: 1.01–1.18), being a female (IRR = 1.10, 95% CI: 1.01–1.20), high subjective socioeconomic status (SES) [IRR = 1.11, 95% CI: 1.02–1.21], and skipping tooth-brushing (IRR = 1.09, 95% CI: 1.00–1.19) were associated with a higher DMFT index. DMFT index also increased among students who reported regular dental visits (IRR = 1.22, 95% CI: 1.10–1.36), but their odds of being in the dental caries-free group decreased (odds ratio [OR] = 0.38, 95% CI: 0.18–0.82). Significant predictors of being categorised to the SiC group were older age (OR = 1.41, 95% CI: 1.03–1.92), high subjective SES (OR = 1.57, 95% CI: 1.13–2.19), and regular dental visits (OR = 2.34, 95% CI: 1.56–3.51).

**Conclusions:**

A high prevalence of dental caries and high DMFT index, with a dominance of FT, were observed in our Russian medical and dental students. Age, sex, subjective SES, regular dental visits, and skipping tooth-brushing were determinants of dental caries experience.

## Background

Dental caries is a widespread chronic disease that affects billions of people worldwide. In the last decades, marked improvements in dental health have been reported in developed countries, along with an increasing proportion of dental caries-free populations, likely due to the implementation of preventive programmes such as water fluoridation, introduction of fluoride in toothpaste, and positive changes in oral health behaviour [1]. Nonetheless, global problems related to dental caries persist in most industrialised countries. The prevalence of dental caries ranges from 60 to 90% in schoolchildren and is almost 100% in adults [2]. According to the World Health Organisation (WHO), children aged 12 years are a key group that need to be monitored for dental caries. Dental caries experience at this age, expressed using the decayed (D) missing (M) filled (F) Teeth (T) index, varies from 0.2 to 7.8 across countries [3]. In Russia, the prevalence of dental caries (DMFT >0) is still high; considerably higher than in neighbouring Nordic countries. In 2009, the proportion of 12-year-olds with no dental caries experience (DMFT = 0) was 52% in Norway and 16% in Russia [4].

Young adults aged 18–25 years are also a particularly important group in the study of dental health and its determinants. Indeed, this age range comprises periods of biological, psychological, and social development and is a transition between adolescence and adulthood, when persons take responsibility for their health and develop their own health behaviour [5]. Conscripts and students are often targeted in studies of dental health in young adults, and previous studies in these populations have been conducted in many countries, including Japan [5], Israel [6], Brazil [7], Norway [8], Australia [9, 10], Finland [11], and China [12]. Previously reported risk factors associated with dental health include socioeconomic factors (income, education, occupation) [7, 10, 11], socio-demographic factors (age, sex, place of residence, ethnicity) [5, 10, 11], oral health behaviour and attitudes [6, 12], and exposure to fluoridated drinking water [10, 11, 13].

However, to our knowledge, there is little information on dental caries experience and determinants in young adults in Russia. In 2006–2008, a group of researchers conducted a study among 432 students in Moscow aged 16–25 years. They reported a high mean DMFT index (10.4) and mean DT (5.7), and the reported prevalence of dental caries was 100% and 98.3% in females and males, respectively [14]. An epidemiological survey from the Arkhangelsk Region of North-West Russia investigated the dental health of 447 conscripts aged 18-19 years and reported a prevalence of dental caries of 94.3% and a mean DMFT of 5.9 [15]. However, both of these studies presented dental status in a descriptive manner; no determinants were studied.

Medical and dental students are expected to have specific knowledge about disease prevention and hygiene, and thereby are expected to show better oral health behaviour compared to their counterparts in the general population. In addition, students from medical and dental faculties may have high socioeconomic status (SES), which in turn may lead to better dental health [16]. Nevertheless, in 2008, an Indian study revealed that only 54.6% and 38.5% of the dental and medical students, respectively, brushed their teeth twice a day. Moreover, more than 80% of the study participants had never used dental floss [17]. In Russia, there is only one study that examined medical students, which was performed in 1987 [18]. The authors observed a high prevalence of dental caries (98.5%) and a mean DMFT of 9.3, reflecting poor oral health.

The present study aimed to investigate dental caries experience and determinants in medical and dental students in North-West Russia.

## Methods

The Northern State Medical University (NSMU) is located in Arkhangelsk, Russia. Students at the NSMU are mainly from the European North-West of Russia, which includes the regions of Arkhangelsk, Vologda, Murmansk, the Komi Republic, the Republic of Karelia, and the Nenets Autonomous Okrug. Altogether, these regions cover an area of approximately 1.5 million km^2^ and have a population of 4.6 million (78.9% urban in 2016) [19].

We selected our participants from the approximately 3900 students that attended the NSMU in the 2015-2016 academic year. We invited students from two faculties to participate: 1) the medical faculty, which included students from the departments of general medicine and paediatric medicine; and 2) the dental faculty. For convenience, students from four other, smaller faculties and departments (medical prophylaxis, medical biochemistry, pharmacy, and clinical psychology) were not considered; nor were students from the international faculty of general practitioners, as we focused on students of Russian nationality only.

This cross-sectional study included two stages. At Stage 1, students from in both faculties and all years of education (6 years for medical students and 5 years for dental students) were informed about the study and invited to participate at the end of a scheduled curriculum classroom lecture. Altogether, 1579 students attended this lecture and were invited to Stage 1. The overall attendance rate of the lectures was 78.7% and varied from 55.1% (6th-year medical students) to 100% (4th-year medical students). Of the invited students, 1385 agreed to participate (overall response rate 87.7%). During the last 15 min of the lecture, they signed the informed consent form and completed a structured, self-administered, anonymous questionnaire in Russian under the supervision of the main researcher (SND). The response rates were similar across the faculties and years of education (>83.3%), except for 4th-year medical students (57.8%). All students participating in Stage 1 gave their mobile phone number so they could be contacted for Stage 2.

**Fig. 1:**
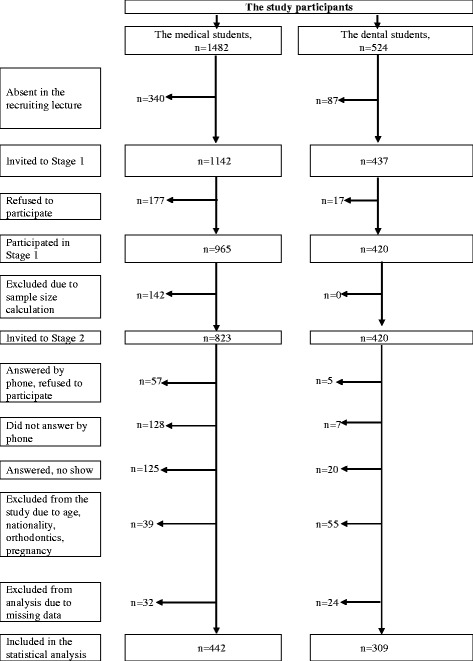
Flow chart of the study participants

Stage 2 included the completion of a second, structured, self-administered, anonymous questionnaire and a clinical dental examination. In order to get comparable groups of medical and dental students and taking into account an outcome prevalence of 0.50, a confidence interval (CI) of 95%, and error margin of 5%, the necessary sample size was calculated as ~380 students in each group. Assuming that medical students may not be as supportive of the oral health study as dental students, and allowing for refusals, no-shows, and exclusions, we invited 420 dental students and 823 medical students to attend Stage 2. For medical students, a stratified random proportionate sample was selected, taking into consideration the distribution of medical students across the departments (general medicine and paediatric medicine) and years of education. Sixty-two students (57 medical and 5 dental) refused to participate in Stage 2 after invitation. We excluded 135 students (128 medical and 7 dental) who did not answer their phone at two separate calls on two separate days and 145 students (125 medical and 20 dental) who did not attend the clinical dental appointment. Ninety-four students (39 medical and 55 dental) were also excluded due to the exclusion criteria for the clinical dental examination (age under 18 or over 25 years, non-Russian nationality, presence of fixed orthodontics bands, and pregnancy). The response rate was 57.6% and 79.3% in medical and dental students, respectively, and varied across years of education (41.5-69.1% and 70.3–85.4%, respectively). Finally, 56 students with missing data in the questionnaires were excluded. Thus the final sample for analysis consisted of 442 medical and 309 dental students (Fig. 1).

### Data collection

The Stage 1 questionnaire collected information on socio-demographic variables, as well as data on oral health behaviour. Age was categorised as 18–20 and 21-25 years in order to get results that could be compared with those of other studies. Other socio-demographic variables included sex, faculty (medical/dental), childhood place of residence (urban/rural), and location of finishing school (Arkhangelsk City/Arkhangelsk Region/other regions). The questionnaire also asked the student to report whether they were eligible for free education (no/yes) and their type of accommodation (hostel/flat or house). A university applicant who does not qualify for free education at the NSMU can still study there, but they must pay tuition each year.

Questions on oral health behaviour included frequency of tooth-brushing (infrequent, i.e., never/less than once a week/once every few days/once a day; or frequent, i.e., twice a day/more than twice a day), using toothpaste with fluoride (without fluoride/difficult to answer; or with fluoride), and skipping tooth-brushing (no, i.e., never or almost never; and yes, i.e., sometimes during a week/every day or almost every day). Regularity of dental visits was categorised as regular (at least once every 6 months/at least once a year) and not regular (occasionally/no visits during the last 3 years). The option ‘difficult to answer’ was chosen only twice in response to regularity of dental visits and thus was considered as missing in the analysis.

The Stage 2 questionnaire collected additional information on socio-demographic variables. Mother’s education was categorised as lower than university (high school: 9–11 years of school; specialised secondary: professional, medical, or pedagogical college, technicum) and university. The response ‘difficult to answer’ was considered as a missing value. Subjective SES was assessed using the MacArthur Scale [20], in which students self-reported the ranking of their family in Russian society on a ladder with 10 rungs in accordance with socioeconomic indicators (education, income, occupation): 10 was ‘best off’ and 1 was ‘worst off’. Given the skewed distribution of SES and using the median SES (6.0) as the cut-off, those who gave a rating of 1–5 were categorised as having low subjective SES and those responding 6–10 as having high subjective SES.

The authors developed the questionnaire in English and two independent persons translated/back-translated to Russian. The final versions were discussed and were judged to concur with the original. Before the study began, the questionnaires were tested on 12 students randomly selected from the target age group who did not participate in the study. No adjustments were necessary.

A non-invasive clinical dental examination was performed at the Dental Clinic of the NSMU from February to May 2016. The students were examined in a dental chair under a professional light, using a dental plain mirror and a dental probe without radiographs. One researcher (SND) executed all clinical examinations and dictated observations to an assistant in the room, who recorded them on a clinical form. Clinical criteria for dental caries were applied in accordance with WHO recommendations (i.e., when a lesion of the tooth’s surface had an unmistakable cavity, undermined enamel, or a detectably softened floor or wall) [21]. All permanent teeth, excluding wisdom teeth, were taken into consideration during the clinical examination. The researcher was carefully calibrated on examination technics and diagnostic thresholds at the Dental Clinic of UiT The Arctic University of Norway, Tromsø, Norway, before the study start. Information from the dental clinical examination was used to calculate DMFT index. The Significant Caries (SiC) index was computed as the mean DMFT index in the tertile of participants with the highest DMFT index [22]. The DMFT cut-off point in this subgroup was 9, thus all students with a DMFT index ≥9 were placed in the SiC group.

Fifty-four (7%) of the examined students were selected randomly for clinical re-examination in June 2016. To ensure reliability, Cohen’s Kappa and intraclass correlation coefficients (ICC) were calculated for dichotomous and quantitative data, respectively. The Kappa statistic for DT and non DT teeth was 0.804 (95% CI: 0.641-0.967), signifying a strong agreement [23]. For DMFT index, the ICC was 0.989 (95% CI: 0.981–0.993).

### Statistical analysis

Data analysis was performed with IBM SPSS Statistics for Macintosh version 23.0 (IBM Corp., Armonk, New York, USA) and STATA version 14.0 (StataCorp, College Station, Texas, USA). Given the skewed distribution of the DMFT index, the Mann-Whitney U and Kruskal-Wallis tests were used to compare two and more than two independent groups, respectively. For qualitative data, the chi-square test was applied.

The Poisson model, the negative binomial model, the zero-inflated (ZI) models, and the hurdle models were taken into consideration to explore the effects of socio-demographic factors and oral health behaviour on DMFT index. The significant likelihood-ratio test of alpha (chi-square = 267.2, *p* < 0.001) indicated that the data were over-dispersed and that negative binomial regression fitted the data better than Poisson regression. Moreover, the significant Vuong test (z = 3.10, *p* = 0.001) showed an excessive number of zeros. For outcome distributions with over-dispersion and an excess of zeros, the zero-inflated negative binomial model or the negative binomial hurdle (NBH) model are recommended [24]. Both ZI and hurdle models consist of a zero part and a count distribution part. In ZI models, zeros can be specified in either the zero part (structural zeros) or in the count distribution part (sampling zeros), which often leads to an incorrect or imprecise interpretation of the results [25]. In contrast, the two parts of hurdle models are clearly separated, and all zeros are modeled only in the zero part, while the count part (or zero-truncated part) deals with values over zero. For this reason, hurdle models have an easier and less misleading interpretation [24]. Therefore, we applied NBH analysis that included two separate models: a logistic regression and a zero-truncated negative binomial regression. The first model predicts whether or not a student experiences dental caries (i.e., DMFT >0 vs. DMFT = 0). The second model was generated to predict the DMFT index for students with dental caries experience. Two sets of predictors were used for different parts of the NBH regression model. The selection of variables included in logistic and zero-truncated parts of the NBH model was determined by their level of significance (less than 0.2) in univariable analysis for the proportion of dental caries-free students (DMFT = 0) and mean DMFT index (DMFT >0), respectively. To adjust for heterogeneity, Huber-White sandwich estimates for standard errors (robust estimates) were applied.

In addition to the NBH analysis, we used multivariable binary logistic regression to evaluate the odds ratios (OR) of being categorised to the SiC group in relation to selected socio-demographic and oral behavioural determinants. Whether a student was in the SiC group or not was considered as the dependent variable. All variables with a level of significance less than 0.2 in the univariable analysis were included in the multivariable regression model simultaneously. The level of significance for testing all statistical hypotheses was set at *p* = 0.05.

## Results

A total of 751 students were included in the statistical analysis, and the majority were women (*n* = 564). Mean age of the students was 20.2 years (standard deviation [SD] 1.6). Seventy-two percent of the participants reported an urban childhood area of residence, and more than 80% of the students came from the Arkhangelsk Region or other regions of North-West Russia. Almost 80% of the participants were eligible for free education and 64% lived in flat or house. The mothers of 45% of the participants had an education level that was lower than university. When looking at oral health behaviour, 78% of participants reported regular dental visits, 47% reported using a toothpaste with fluoride, and 81% reported frequent tooth-brushing. However, 34% of the students reported skipping tooth-brushing once a week, every day, or almost every day.

**Fig. 2:**
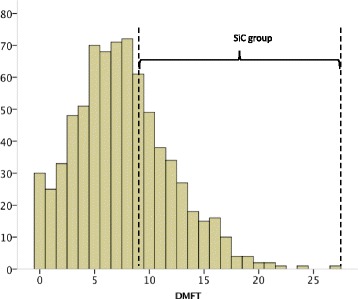
Histogram of the Decayed Missing Filled Teeth (DMFT index) in the overall study sample (*n* = 751) and in the Significant Caries (SiC) group (*n* = 283)

The prevalence of dental caries (DMFT >0) among the participants was 96.0%. The overall mean DMFT index was 7.58 (SD 4.4); DT: 0.61 (SD 1.2), MT: 0.12 (SD 0.4), and FT: 6.84 (SD 4.1), with FT accounting for 90.2% of dental caries experience. FT constituted the main fraction of the DMFT index, both in medical (89.8%) and dental (91.0%) students. The SiC index was 12.50 (SD 3.0); DT: 0.99 (SD 1.5), MT: 0.26 (SD 0.6), and FT: 11.25 (SD 2.9), with FT accounting for 90.0%. There were 283 students (37.7%) in the SiC group (DMFT ≥9) (Fig. 2).

There were no statistically significant differences in the prevalence of dental caries across the socio-demographic characteristics considered. The mean DMFT in students with dental caries experience and the proportion of students in the SiC group (DMFT ≥9) were significantly higher among participants aged 21-25 years than among their younger counterparts (8.3 vs. 7.6 and 43% vs. 34%, respectively). Students with dental caries experience who were not eligible for free education had a lower DMFT index compared to those who were eligible for free education. Students with higher subjective SES had a significantly higher DMFT index (8.2 vs. 7.4) and presented in the SiC group more frequently (41% vs. 31%). No statistically significant differences in the mean DMFT index and proportion of students in the SiC group were observed across age, sex, faculty, place of childhood residence, location of finishing school, accommodation, and level of mother’s education (Table 1).

**Table 1:** Socio-demographic characteristics associated with dental caries experience in the study sample

	All n	DMFT = 0 (%)	p*	DMFT >0, mean (SD)	p**	SiC gr n (%)	p*
Age group (years)			0.054		0.028		0.020
18–20	449	(5.1)		7.62 (4.10)		154 (34.3)	
21–25	302	(2.3)		8.30 (4.33)		129 (42.7)	
Sex			0.657		0.053		0.068
Male	187	(4.8)		7.31 (3.79)		60 (32.1)	
Female	564	(3.7)		8.09 (4.32)		223 (39.5)	
Faculty			0.611		0.062		0.149
Medical	442	(4.3)		8.12 (4.26)		176 (39.8)	
Dental	309	(3.6)		7.58 (4.13)		107 (34.6)	
Place of childhood residence			0.398		0.725		0.821
Urban	537	(4.5)		7.91 (4.18)		201 (37.4)	
Rural	214	(2.8)		7.86 (4.30)		82 (38.3)	
Location of finishing school			0.547		0.951		0.485
Arkhangelsk City	146	(5.5)		7.92 (4.56)		49 (33.6)	
Arkhangelsk Region	302	(3.3)		7.84 (4.09)		119 (39.4)	
Other regions of North-West Russia^a^	303	(4.0)		7.94 (4.17)		115 (38.0)	
Eligible for free education			0.408		0.016		0.164
Yes	593	(4.4)		8.07 (4.17)		231 (39.0)	
No	158	(2.5)		7.26 (4.29)		52 (32.9)	
Subjective SES			0.598		0.013		0.005
Less than 6.0	259	(3.5)		7.36 (3.97)		80 (30.9)	
6.0 and more	492	(4.3)		8.18 (4.31)		203 (41.3)	
Accommodation			0.149		0.454		0.345
Hostel	268	(2.6)		7.80 (4.15)		107 (39.9)	
Flat/house	483	(4.8)		8.05 (4.31)		176 (36.4)	
Mother’s education			0.084		0.095		0.199
Lower than university	341	(2.6)		7.70 (4.42)		120 (35.2)	
University	410	(5.1)		8.06 (4.02)		163 (39.8)	

Abbreviations: *DMFT* Decayed Missing and Filled Permanent Teeth, *SD* Standard Deviation, SiC *gr* Significant Caries group, *SES* socioeconomic status

^a^Vologda Region, Komi Republic, Murmansk Region, Republic of Karelia or Nenets Autonomous Okrug

* *p*-value from the Chi square test; **p-value from the Mann-Whitney U test for two independent groups and the Kruskal-Wallis test for three independent groups

Students who reported regular dental visits had a higher prevalence of dental caries and a higher DMFT index, fewer DT (0.56 vs. 0.81, *p* = 0.025), more FT (7.28 vs. 5.33, p<0.001), and were more frequently in the SiC group compared to those who did not report such visits. No statistically significant differences were found in the prevalence of dental caries or the DMFT index among categories of tooth-brushing, skipping tooth-brushing, or toothpaste (Table 2).

**Table 2:** Oral health behaviours associated with dental caries experience in the study sample

	All n	DMFT = 0 (%)	p*	DMFT >0, mean (SD)	p**	SiC gr n (%)	p*
Regularity of dental visits			0.030		<0.001		<0.001
Regularly	584	(3.1)		8.21 (4.18)		244 (41.8)	
Not regularly	167	(7.2)		6.75 (4.13)		39 (23.4)	
Tooth-brushing			0.723		0.904		0.532
Infrequent	144	(4.9)		7.74 (4.09)		51 (35.4)	
Frequent	607	(3.8)		7.93 (4.24)		232 (38.2)	
Toothpaste			0.122		0.159		0.334
Without fluoride or difficult to answer	397	(5.0)		8.12 (4.27)		156 (39.3)	
With fluoride	354	(2.8)		7.64 (4.13)		127 (35.9)	
Skipping tooth-brushing			0.100		0.179		0.347
No	496	(4.8)		7.74 (4.19)		181 (36.5)	
Yes	255	(2.4)		8.18 (4.23)		102 (40.0)	

Abbreviations: *DMFT* Decayed Missing and Filled Permanent Teeth, *SD* Standard Deviation, SiC *gr* Significant Caries group

*p-value from the Chi square test; **p-value from the Mann-Whitney U test for two independent groups

The results of NBH are shown separately for the logistic and zero-truncated negative binomial parts (Table 3). Regular dental visits were significantly associated with lower odds of being in the dental caries-free group (OR = 0.38, 95% CI: 0.18–0.82). Furthermore, students who reported regular dental visits had an adjusted DMFT index that was 1.22 (95% CI: 1.10–1.36) times higher than that observed in those who did not report such visits. The DMFT index of students aged 21–25 years was 1.09 (95% CI: 1.01–1.18) times higher than that predicted in their younger counterparts, after adjustment for other variables in the model. Being a female, skipping tooth-brushing, and high subjective SES were also found to be significant independent determinants of high DMFT index.

**Table 3:** Association between the DMFT index and selected determinants in the negative binomial hurdle model

Determinants^a^	Logistic regression^b^		Zero-truncated negative binomial regression^c^	
	OR (95% CI)	*p*-value	IRR (95%CI)	*p*-value
Age group (years)		0.164		0.031
21–25	0.52 (0.21–1.30)		1.09 (1.01–1.18)	
18–20	Reference		Reference	
Sex				0.037
Female			1.10 (1.01–1.20)	
Male			Reference	
Faculty				0.283
Dental			0.95 (0.88–1.04)	
Medical			Reference	
Eligible for free education				0.172
No			0.93 (0.83–1.03)	
Yes			Reference	
Subjective SES				0.015
6.0 and more			1.11 (1.02–1.21)	
Less than 6.0			Reference	
Accommodation		0.361		
Hostel	0.65 (0.25–1.64)			
Flat or house	Reference			
Mother’s education		0.093		0.287
University	1.99 (0.89–4.42)		1.04 (0.96–1.13)	
Lower than university	Reference		Reference	
Skipping tooth-brushing		0.061		0.047
Yes	0.41 (0.16–1.04)		1.09 (1.00–1.19)	
No	Reference		Reference	
Toothpaste		0.143		0.117
With fluoride	0.56 (0.26–1.22)		0.94 (0.87–1.02)	
Without fluoride or difficult to answer	Reference		Reference	
Regularity of dental visits		0.013		<0.001
Regularly	0.38 (0.18–0.82)		1.22 (1.10–1.36)	
Not regularly	Reference		Reference	

Abbreviations: *DMFT* Decayed Missing and Filled Permanent Teeth, *IRR* incidence rate ratio, *CI* confidence interval, *OR* odds ratio, *SES* socioeconomic status

^a^Accommodation was included only in the logistic regression. Sex, Faculty, Eligible for free education, and Subjective SES were included only in the zero-truncated negative binominal regression

^b^The dependent variable was whether a student was dental caries-free (coded as 1) or not (coded as 0)

^c^The dependent variable was the count zero-truncated variable (DMFT >0)

Significant predictors of being categorised to the SiC group were older age (OR = 1.41, 95% CI: 1.03–1.92), high subjective SES (OR = 1.57, 95% CI: 1.13–2.19), and regular dental visits (OR = 2.34, 95% CI: 1.56–3.51) (Table 4).

**Table 4:** Adjusted odds ratio of being in the Significant Caries group for selected determinants

Determinants^a^	Adjusted OR (95% CI)	*p*-value
Age group (years)		0.030
18–20	Reference	
21–25	1.41 (1.03–1.92)	
Sex		0.271
Male	Reference	
Female	1.23 (0.85–1.77)	
Faculty		0.120
Medical	Reference	
Dental	0.77 (0.56–1.07)	
Subjective SES		0.008
Less than 6.0	Reference	
6.0 and more	1.57 (1.13–2.19)	
Eligible for free education		0.412
Yes	Reference	
No	0.85 (0.57–1.26)	
Mother’s education		0.308
Lower than university	Reference	
University	1.18 (0.86–1.61)	
Regularity of dental visits		<0.001
Not regularly	Reference	
Regularly	2.34 (1.56–3.51)	

Abbreviations: *DMFT* Decayed Missing and Filled Permanent Teeth, *CI* confidence interval, *OR* odds ratio, *SES* socioeconomic status

^a^Results from the multivariable binary logistic regression; all listed variables were included in the model simultaneously; p-value of the Hosmer-Lemeshow goodness of fit test =0.474; Negelkerke R square = 6.9%

## Discussion

Our study showed high dental caries prevalence and high dental caries experience with dominance of FT among undergraduate medical and dental Russian students aged 18–25 years in North-West Russia. Age, sex, subjective SES, skipping tooth-brushing, and regular dental visits were found to be significant determinants of DMFT index.

This is the first study in North-West Russia in almost 20 years to investigate dental caries experience and its determinants in young adults aged 18–25 years. The dental health status reported in this study was based on clinical dental examination and reliability tests showed the consistency of the obtained data. The overall response rate was quite high: 87.7% and 64.9% for Stages 1 and 2, respectively.

However, this study does have some limitations. Firstly, due to its cross-sectional design, this study does not allow us to evaluate causal relationships, risk of dental caries development, or trends in the prevalence of dental caries and dental caries experience over time. Secondly, we included only medical and dental students from the NSMU; therefore the generalisability of the results to other young adults may be questioned. We assume that medical and dental students are, to some extent, a prosperous group of young people with regard to SES and health-related issues, including dental health. However, the participants reported a subjective SES of regular/good (median of MacArthur scale is 6.0), indicating that they perceived themselves to belong to a group not far from the average. On the other hand, information on SES and oral health behaviour in the present study was self-reported; thus the possibility of bias due to under- or over-reporting cannot be excluded. Thirdly, only visual and tactile methods were applied for dental caries detection; radiographs were not taken, which could lead to an underestimation of dental caries.

The prevalence of dental caries among medical and dental students in the present study (95.7% and 96.4%) was higher than that reported in Spain (82.2% and 83.0% at the start and 91.1% and 87.2% at the end of study) [26] and in Yemen (81.7% and 85.0%) [16]. A similar pattern was observed in relation to the extent of dental caries experience, as measured by the high mean DMFT index of 7.6, which shows that the dental health of medical and dental students in North-West Russia is worse than that reported in Spain [26], India [17], and Yemen [16]. We did not find differences in the DMFT index of medical and dental students, which is in contrast with other studies. In 2002, Spanish researchers performed a longitudinal study and reported that medical students had a lower DMFT index than dental students: 3.4 vs. 5.0 in the third year, and 4.3 vs. 5.9 in the fifth year of education [26]. In contrast, an Indian study found a mean DMFT index of 1.2 in dental students vs. 2.0 in medical students [17]. Nevertheless, in 2008–2009, Halboub et al. examined a sample of students from the faculties of medicine, dentistry, and literature at Sana’a University, Yemen, and also found no statistically significant differences in overall DMFT index between the faculties (3.9, 4.3, and 4.2, respectively) [16]. Our finding may be explained by the fact that dental caries is a slow disease and its development may start long before persons decide on dental or medical education.

Other Russian studies among Perm medical students aged 19–20 years and students from Moscow aged 21-25 years found that only 1.5% [18] and 0.7% [14] were dental caries-free, respectively. In these Russian studies, published in 1987 [18] and 2009 [14], the DMFT index was even higher than ours: 9.3 and 10.4, respectively. Direct comparison of these results with our data must be done with caution due to differences in population characteristics, recruitment of the participants, and the area covered. Nonetheless, one may speculate that dental health in young adults in Russia has not significantly improved despite positive socio-economic changes in Russia over the past 30 years.

In the current study, FT constituted the main fraction of the DMFT index, both in medical (89.8%) and dental (91.0%) students. This fraction was very high compared to medical and dental students from India (21.4% and 34.5%) [17] and Yemen (54.6% and 49.9%) [16]. A Spanish study reported that FT accounted for 60.4% and 56.4% of dental caries experience in 3rd- and 5th-year medical students, respectively. In contrast, the FT fraction in dental students constituted 81.5% of the DMFT index at the start of the study and 88.5% and at the end of the study, reflecting that dental students received more dental treatment than their medical peers [26]. Other Russian studies revealed that FT scores constituted only 42.0% and 60.7% of the DMFT index in students studying in Moscow [14] and in medical students in Perm [18], respectively. High availability of dental treatment and willingness of our medical and dental students to seek such care is one possible explanation for the high fraction of FT in the DMFT index in our study sample. Indeed, in the current study 77.8% of the students reported regular dental visits.

In agreement with the world trend, the DMFT index in the present study increased significantly with age, as dental caries is an irreversible, accumulative disease. According to previous international findings, women tend to have a higher DMFT index than men [5, 27, 28]. In our study, we also found sex differences in the DMFT index in multivariable analysis. Researchers explain this fact through a complex aetiology, including hormonal fluctuations, genetic variations, different saliva composition and flow rate, dietary habits, and social roles in the family [29, 30].

Oral health inequalities associated with SES are widely observed, as persons with low SES have a higher risk of poor dental health in terms of dental caries [31]. We found the opposite association both in the univariable and multivariable analyses, as those with higher subjective SES had a higher DMFT index and were more likely to be in the SiC group. One possible explanation for our findings may be that students with higher SES tend to adapt more to a Westernised lifestyle, with frequent consumption of foods and beverages containing added sugar. Moreover, these students may seek dental treatment more often, as they have less concerns about cost. Nevertheless, as we used self-reported measures of SES, our results might be biased compared to other studies that used education, occupation, or income as more objective indicators of SES.

The importance of oral health behaviour in maintaining good oral and dental health is well established. In our study, 80.8% of the medical and dental students reported brushing their teeth twice a day or more. This is higher than the percentage reported for the past 5-10 years in university students from 26 countries across Asia, Africa, and the Americas (67.2%) [32], Turkish dental students (49.7%) [33], Yemen students (38.1%) [16], and Indian medical students (24.4%) [34]. Nevertheless, the dental health of our study participants was worse than that reported in the aforementioned studies. Over-reporting of good dental behaviour by the participants, given their educational background, cannot be excluded. The fact that 34% of the students reported skipping tooth-brushing and the lack of significant differences in mean DMFT index by tooth-brushing frequency support this assumption, as do the results of the multivariable analysis: skipping tooth-brushing was a significant determinant of higher DMFT index.

Our finding that those who visit a dentist regularly have a higher DMFT is in agreement with previous Chinese [12] and Australian [35] studies. The fact that more than 90% of DMFT in our study were FT may suggest that dental services in Russia are focused on treatment, not on dental caries prevention.

The SiC index was introduced to focus on persons with the highest DMFT index and to solve the problem of a skewed dental caries distribution [22]. We did not find any publications on SiC index among medical and dental students in Russia or other countries that can be compared with our results. Nevertheless, the SiC index of 12.5 (with FT accounting for 90.0%) in our study reflects a high number of students with a high DMFT index (with high FT component). The variables associated with the odds of being categorised to the SiC group (dental visits, subjective SES, age) were the same as those associated with high DMFT index. One may speculate that students have a lack of knowledge regarding a healthy diet and/or appropriate oral hygiene habits, which in turn may lead to frequent dental visits for dental treatment. Further studies that include information on the threshold for dental caries treatment among Russian dentists are warranted to better understand the high DMFT in our study population.

## Conclusions

High dental caries prevalence and high DMFT index, with a dominance of FT, were observed among undergraduate medical and dental Russian students aged 18-25 years in North-West Russia. Age, sex, subjective SES, regular dental visits, and skipping tooth-brushing were found to be significant determinants of dental caries experience.

## Abbreviations

CI: Confidence interval
DMFT: Decayed missing filled teeth
DT: Decayed teeth
FT: Filled teeth
ICC: Intraclass correlation coefficient
IRR: Incidence rate ratio
MT: Missing teeth
NBH: Negative binomial hurdle model
NSMU: Northern State Medical University
OR: Odds ratio
SD: Standard deviation
SES: Socioeconomic status
SiC index: Significant caries index
WHO: World health Organisation
ZI: Zero-inflated model
